# Fractional Talbot Lithography for Predesigned Large-Area Liquid-Crystal Alignment

**DOI:** 10.3390/ma17194810

**Published:** 2024-09-30

**Authors:** Zhichao Ji, Zenghua Gan, Yu Wang, Zhijian Liu, Donghao Yang, Yujie Fan, Wenhua Li, Irena Drevensek-Olenik, Yigang Li, Xinzheng Zhang

**Affiliations:** 1 College of Physics and Electronic Engineering, Xinyang Normal University, Xinyang 464000, China; jizc@xynu.edu.cn; 2 The MOE Key Laboratory of Weak-Light Nonlinear Photonics and International Sino-Slovenian Join Research Center on Liquid Crystal Photonics, TEDA Institute of Applied Physics and School of Physics, Nankai University, Tianjin 300457, China; 2120210201@mail.nankai.edu.cn (Z.G.); 1120190080@mail.nankai.edu.cn (Y.W.); 2120230264@mail.nankai.edu.cn (Z.L.); yangdonghao0305@126.com (D.Y.); 2210369@mail.nankai.edu.cn (Y.F.); liyigang@nankai.edu.cn (Y.L.); 3 Faculty of Mathematics and Physics, University of Ljubljana, SI-1000 Ljubljana, Slovenia; irena.drevensek@ijs.si; 4 Department of Complex Matter, J. Stefan Institute, SI-1000 Ljubljana, Slovenia; 5 Collaborative Innovation Center of Extreme Optics, Shanxi University, Taiyuan 030006, China

**Keywords:** fractional Talbot lithography, liquid-crystal alignment, anchoring energy, *q*-plate, special optical fields

## Abstract

To address the increasing demands for cost-effective, large-area, and precisely patterned alignment of liquid crystals, a fractional Talbot lithography alignment technique was proposed. A light intensity distribution with a double spatial frequency of a photomask could be achieved based on the fractional Talbot effect, which not only enhanced the resolution of lithography but also slashed system costs with remarkable efficiency. To verify the feasibility of the alignment method, we prepared a one-dimensional polymer grating as an alignment layer. A uniform alignment over a large area was achieved thanks to the perfect periodicity and groove depth of several hundred nanometers. The anchoring energy of the alignment layer was 1.82 × 10^−4^ J/m^2^, measured using the twist balance method, which surpassed that of conventional rubbing alignment. Furthermore, to demonstrate its ability for non-uniform alignment, we prepared polymer concentric rings as an alignment layer, resulting in a liquid-crystal *q*-plate with *q* = 1 and $\alpha_{0}$ = π/2. This device, with a wide tuning range (phase retardation of ~6π @ 633 nm for 0 to 5 V), was used to generate special optical fields. The results demonstrate that this approach allows for the uniform large-area orientation of liquid-crystal molecules with superior anchoring energy and customizable patterned alignment, which has extensive application value in liquid-crystal displays, generating special optical fields and intricate liquid-crystal topological defects over a large area.

## 1. Introduction

As a kind of wonderfully soft material, liquid crystals (LCs) have gained the favor of researchers due to their excellent electro-optical properties and sensitive response characteristics to external stimuli. The liquid-crystal devices are typical sandwich structures with liquid-crystal molecules sandwiched between the alignment layers on ITO-coated glass substrates. As the function layer, the aligned liquid-crystal molecules decide the performance of the devices. For traditional displays, the spatial distribution of liquid-crystal directors is typically uniform, so the transmitted light intensity can be modulated easily through electrical tuning [1]. Furthermore, in non-display applications, such as liquid-crystal polarization gratings and topological defects, the orientation of liquid-crystal molecules is non-uniform and programmed, so that the incident light can be tailored precisely [2,3,4]. Driven by the evolving requirements for liquid-crystal functional devices, the key liquid-crystalline alignment technologies for the devices have also undergone significant advancements, including rubbing alignment [5,6], photoalignment [7,8], micro-rubbing [9,10], nanoimprint lithography [11], direct laser writing with two-photon polymerization (DLW-TPP) [12], and patterned electrodes [13].

Based on the Berreman theory [14,15], the periodic geometric structure can effectively induce liquid-crystal molecules to align along the grooves through their depth limitation, forming a stable orientation state, which is also referred to as geometric confinement [16]. Therefore, mature micro/nano-processing technologies, such as nanoimprint lithography [11] and DLW-TPP [12], are gradually being applied in the liquid-crystal field, especially for the micro-zone and complex patterned orientation. However, these technologies generally have a low processing efficiency, high equipment costs, and impossible large-area uniform alignment, just like rubbing alignment [17]. In contrast, traditional photolithography technology exhibits advantages in time and cost for preparing micro/nanostructures, but its resolution highly depends on the cost of the photomasks. Undoubtedly, a more flexible approach to liquid-crystal orientation that can balance a large area, design ability, high efficiency, and low cost remains a challenge for liquid-crystal displays and non-display applications [18].

The Talbot effect, a unique Fresnel diffraction phenomenon, exhibits self-images of a periodic photomask at specific locations under the illumination of collimated light [19]. Therefore, it demonstrates broad application potential in fields such as image processing, nonlinear optics, quantum imaging, and waveguides. In recent years, photolithography technology based on the Talbot effect has achieved significant advancements in both high-precision and large-area applications [20,21,22,23,24,25]. However, as the feature size reduces to the submicron level, the costs of photomasks and extreme ultraviolet light sources will increase significantly, causing a challenge for further development of this technology [26]. In fact, a high resolution is not a mandatory requirement, and near-micrometer-scale patterning also holds practical value in many fields, especially in liquid-crystal alignment [27,28]. More attention should be paid to large-scale production and cost control. Although, using Talbot lithography technology, one can design and fabricate large-area, high-resolution periodic structures, its application in liquid-crystal alignment control is relatively rare, especially in terms of patterned alignment. For example, Yuan et al. achieved sub-micrometer-scale square and hexagonal photonic crystals in the polymer-dispersed liquid-crystal (PDLC) system based on the Talbot effect [29]. Liu et al. successfully prepared PDLC photonic crystals with a lattice constant of approximate 0.9 μm [30]. These studies were all based on the Talbot effect to produce a smaller period-intensity distribution, inducing periodic phase separation in liquid-crystal/polymer composites rather than directly controlling the alignment of liquid-crystal molecules. Therefore, an in-depth exploration of liquid-crystal alignment technology based on the Talbot effect is of great significance for achieving low-cost, large-area, and designable liquid-crystal alignment distributions.

In this paper, we proposed a novel low-cost liquid-crystal alignment technology based on fractional Talbot lithography and demonstrated its feasibility in achieving large-area and designable patterned alignment. An ordinary ultraviolet LED was used as the exposure light source to illuminate a one-dimensional (1D) grating photomask and a two-dimensional (2D) concentric ring photomask, both with a period of 3 μm and a silt width of 1 μm; then, 1D grating and concentric ring polymer films with a period of 1.5 μm could be obtained at the half Talbot distance. Next, the 1D polymer grating films were used as alignment layers to assemble a twisted liquid-crystal cell, exhibiting excellent alignment effects. The anchoring energy was as strong as 1.82 × 10^−4^ J/m^2^, making it highly suitable for achieving a uniform alignment of LCs over large areas. By utilizing the 2D concentric ring films as alignment layers, the non-uniform alignment of LCs was achieved to form a *q*-plate. A nice Maltese cross-pattern could be observed using a polarized optical microscope under a crossed configuration. Meanwhile, it had the advantages of a low driving voltage (~1 V) and a large electrically tunable range (0~5 V, phase retardation ~6π @ 633 nm). By adjusting the polarization state and phase retardation of the incident light, special optical fields, such as axially symmetric vector beams and vortex beams, could be generated. This approach provides a promising avenue for predesigned large-area liquid-crystal alignment, which may have promising applications in liquid-crystal displays and non-display areas.

## 2. Methods

### 2.1. Fractional Talbot Effect

**Figure 1:**
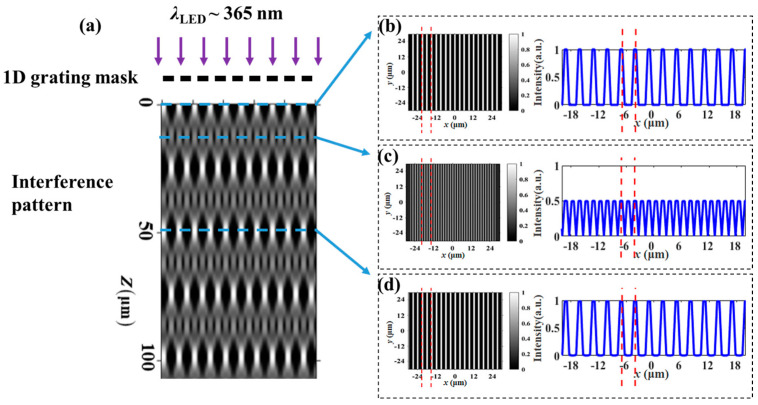
(**a**) Calculated intensity distribution behind a 1D grating photomask; three self-images are marked with blue dashed lines at distances of 0, 12.3 μm, and 49.3 μm from the photomask. The spatial intensity profiles at (**b**) *z* = 0; (**c**) *z* = 12.3 μm = 0.5 *Z*_T_; and (**d**) *z* = 49.3 μm = 2 *Z*_T_, the two red dashed lines indicated the periodic change of the optical field.

As shown in Figure 1a, taking the 1D grating photomask as an example, when a monochromatic plane wave illuminates directly on the photomask, an image of the original grating will be periodically reproduced at different positions behind the mask due to the diffraction effect. This phenomenon is called the integer Talbot effect, and the distance between the photomask and the first image is the integer Talbot distance (*Z*_T_). Besides integer distances, clear periodic images will be easily reproduced at *Z*_T_/*m*, which is referred to as the fractional Talbot distance (*Z_m_*) [19,31]:

(1) $$Z_{m}=\frac{1}{m}\frac{NT^{2}}{\lambda }$$

where *T* is the grating period of the photomask, *λ* is the wavelength of the light source, and *N* and *m* are coprime integers. At the fractional Talbot distance, the grating period of the reconstructed image is 1/*m* of the original period. Therefore, the fractional Talbot effect is often used to improve the resolution of the photolithography system. In theory, the larger *m* is, the smaller the period of the reproduced light field will be. However, the higher *m* will cause a smaller depth of field, which will bring some technical difficulties to lithography. Therefore, it is necessary to achieve a balance between resolution and operability. The appropriate exposure distance is determined through numerical calculation, which serves as the experimental basis for the fractional Talbot lithography to achieve large-area and designable alignment of LCs.

When an ultraviolet light ray is incident on the 1D grating photomask, the optical field distribution along the *z*-direction is shown in Figure 1a, where the periodic light intensity distributions are reproduced at different positions. Figure 1b–d depict the optical field profiles at different Talbot distances marked with the dashed lines in Figure 1a. As shown in Figure 1b,d, there are no changes between the original optical field and that at 2*Z*_T_, exhibiting a high-contrast self-imaging effect. At half of the *Z*_T_, both the intensity and period of the optical field are reduced to half of their original values, and the contrast is somewhat diminished, representing fractional Talbot imaging (*m* = 2). To enhance the experimental feasibility, we selected one-half of the Talbot distance (*m* = 2) as the exposure distance; thus, high-resolution structures can be obtained by using low-resolution photomasks.

### 2.2. Lithography Setup

**Figure 2:**
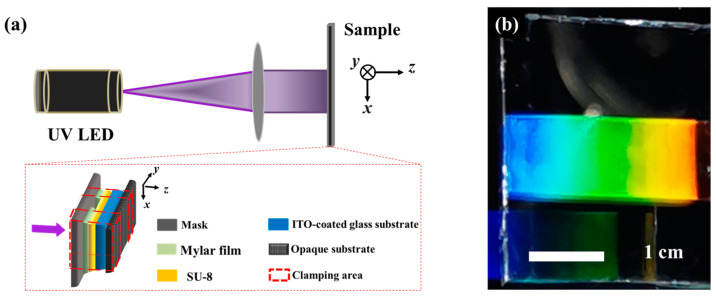
(**a**) Schematic diagram of the lithography setup; the red box is an enlarged view of the sample. (**b**) Photograph of the polymer grating; the colorful region shows the wonderful diffraction by the 1D polymer grating.

The schematic diagram of the UV exposure system is illustrated in Figure 2a. A cost-effective UV-LED with a wavelength of 365 nm and a linewidth of 10 nm (Height-LED Technology Co., Ltd., Shenzhen, China) was employed as the light source. After passing through a convex lens with a diameter of 50.8 mm and a focal length of 500 mm, the collimated light was normally incident on the photomask for exposure processing. Two photomasks (1D grating and 2D concentric ring) used in this work were amplitude masks, tailored by chrome metal as an absorbing layer. To achieve good alignment effects with a low cost, the periodic structures with a grating period of 3 μm and a slit width of 1 μm were designed, respectively. The one-half Talbot distance between the photomask and the sample was controlled by two 12 μm thick Mylar film strips (Beijing Branch, Du Pont China Holding Co., Ltd., Beijing, China) placed along two opposite edges of the gap between the mask and the sample. An opaque substrate with black absorbing material was used to prevent the back reflection. Consequently, this system enabled high-resolution and low-cost periodic structure processing without the need for complex and expensive projection optics, laser sources, or precision positioning systems.

The alignment film used in our experiments was a diluted SU-8 photoresist (SU-8/cyclopentanone with a volume ratio of 1:6), and the SU-8 3010 was purchased from MicroChem Corp., Newton, MA, USA. Before exposure, the photoresist was spin-coated on an ITO-coated glass substrate at a speed of 500 rpm for 6 s and 3000 rpm for 30 s, consecutively. After soft baking at 95 °C for 1 min to evaporate the solvent and densify the film, the substrate was covered with a 400 nm thick layer of photoresist. Then, using the lithography setup in Figure 2a, the photoresist layer was exposed through a photomask for 64 s, and the exposure power was approximately 100 mW. Post-exposure baking was then conducted at 95 °C for 3 min to ensure complete polymerization in the exposed area. The unpolymerized portion was fully dissolved by soaking in the developer for 1 min. A uniform large-area 1D polymer grating (~1 cm × 2.5 cm) was fabricated, as shown in Figure 2b. It can be observed that the structure exhibits dazzling colors under daylight, indicating an excellent periodicity of the polymer structure and the high contrast of the refractive indices between air (*n* = 1) and the SU-8 polymer film (*n* = 1.57). The LC used in this experiment was a nematic LC mixture (E7, Nanjing Ningcui Optical Technology Co., Ltd., Nanjing, China; *n_e_* = 1.741, *n_o_* = 1.517, (@589 nm, 20 °C), T_c_ = 59 °C), and its elastic constants *K*_11_, *K*_22_, and *K*_33_ were 11.7 pN, 5.5 pN, and 14 pN, respectively [32]. When the E7 was filled in the assembly cell with the polymer film, the contrast of the refractive indices was greatly reduced and so was the diffraction.

## 3. Results and Discussion

### 3.1. Fractional Talbot Lithography for Large-Area Uniform Orientation of Liquid-Crystal Molecules

#### 3.1.1. Atomic Force Microscope (AFM) Characterization

Based on the Berreman’s model [14,15], in case of one-elastic-constant approximation (*K*_11_ = *K*_22_ = *K*_33_ = *K*) for LC elastic energy, the anchoring energy of the alignment layer with sinusoidally modulated surface topography can be evaluated qualitatively using Equation (2) [12,33],

(2) $$W_{\varphi B}=\frac{4\pi^{3}KA^{2}}{\Lambda^{3}}$$

**Figure 3:**
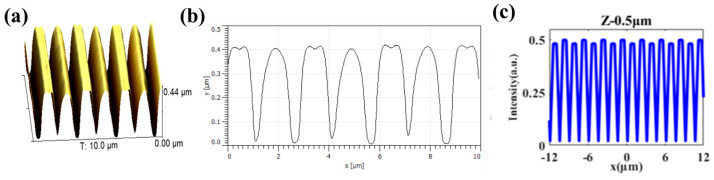
(**a**) AFM morphology of the polymer grating with 1.5 μm periodicity; (**b**) contour of the cross-section along the direction of the grating vector; (**c**) simulated light intensity distribution at a deviation of 0.5 μm from the half Talbot distance.

Obviously, for a chosen LC material, the anchoring energy is related to the groove depth *A* and the groove period *Λ*. As the groove depth increases and the period decreases, the anchoring energy increases accordingly. As shown in Figure 3a, the fabricated 1D polymer grating is characterized by an AFM, which exhibits excellent periodicity through the control of exposure intensity and the distance between the sample and the photomask. From the cross-sectional view of the grating in Figure 3b, it can be seen that the grating structure consists of periodic grooves with a period of 1.5 μm and a depth of 400 nm, and the period is consistent with the intensity distribution at the half Talbot distance analyzed above. Here, a UV-LED with a linewidth of 10 nm was used rather than a laser source. Based on Equation (1), as *Z*_m_ is inversely proportional to the wavelength of the incident light, the wide linewidth can, in principle, form a smeared self-image, which is caused by the overlapping of self-images corresponding to different wavelengths [34]. However, from the results shown in Figure 3a,b, one can see that the 10 nm linewidth UV-LED is acceptable for the fractional Talbot lithography. The polymer grooves have a parabolic-like profile, mainly due to polymerization in the opaque areas caused by diffraction effects [26]. Additionally, periodic variation in the width appears at the top of the polymer grating, primarily due to the alternating changes in light intensity distribution when the distance between the sample and the photomask deviates from one-half of *Z*_T_ [35,36]. As shown in Figure 3c, when the distance between the photomask and sample deviates 0.5 μm from the half Talbot distance, the calculated spatial intensity profile still keeps a 1.5 μm period, and the maximum light intensity changes periodically. During the exposure process, the stronger light will produce enough photo-initialized acid to polymerize the SU-8, resulting in expanded structures on top of the polymer grooves [37]. Furthermore, different expansion coefficients of SU-8 resin and cross-linked SU-8 will cause a tooth-like profile after the post-exposure baking and developing process [38], especially in a stronger light zone.

#### 3.1.2. Polarization Optical Microscopic Images of Twisted Nematic Liquid-Cystal Cells

**Figure 4:**
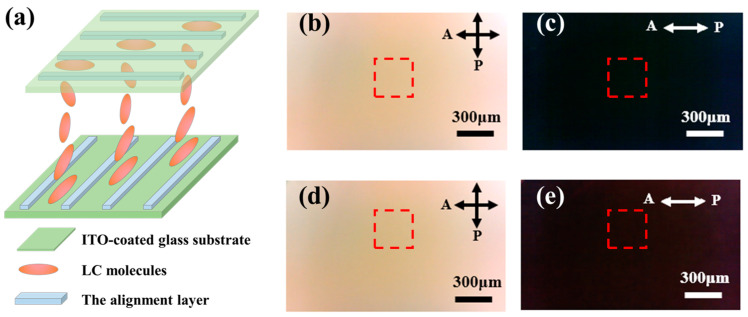
(**a**) Schematic illustration of the TN cells; TN90-1.5 cell: transmission POM images (**b**) under crossed configuration and (**c**) under parallel configuration; TN90-3.0 cell: transmission POM images (**d**) under crossed configuration and (**e**) under parallel configuration, the red boxes indicate the areas chose to calculate the relative intensity.

To investigate the actual anchoring effect of the 1D periodic polymer grating, we assembled two 90° twisted nematic (TN) liquid-crystal cells with a gap of 5 μm, using polymer films with different periods as alignment layers, respectively, and the structural configuration was shown in Figure 4a. One cell (TN90-1.5) adopted alignment layers with a period of 1.5 μm on both the top and bottom substrates, while the other (TN90-3.0) used alignment layers with a period of 3.0 μm obtained using the same photomask with zero distance between the photoresist and the photomask. After filling with LCs, it was observed that under the induction of the upper and lower orientation layers, the directors of the liquid-crystal molecules achieved a continuously and uniformly twisted orientation. The twisted arrangement of the liquid-crystal molecules resulted in a corresponding rotation of the polarization direction of the incident linearly polarized light. As can be seen from the polarization optical microscopic (POM) images in Figure 4b–e, the fields were bright in the crossed configuration and dark in the parallel configuration. Specifically, in Figure 4b–e, the relative intensity in the red box in Figure 4b (189.6) was higher than that in Figure 4d (180.6), and the relative intensity in Figure 4c (13.4) was lower than that Figure 4e (17.3). Namely, the TN90-1.5 cell was significantly darker than the TN90-3.0 cell under a parallel configuration and brighter under a crossed configuration, indicating that the anchoring effect of the polymer film with a 1.5 μm period was better.

#### 3.1.3. Anchoring Energy

**Figure 5:**
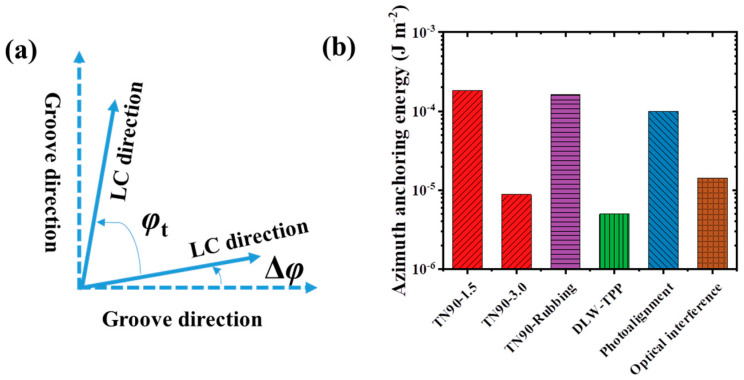
(**a**) Diagram of the groove directions and twisting angles on the inner surfaces of the upper and lower substrates of a 90° twisted liquid-crystal cell; (**b**) anchoring energies of different orientation techniques.

The anchoring energy determines the performance of liquid-crystal devices. To quantitatively study the anchoring energy, we measured liquid-crystal cells composed of three different kinds of orientation layers: TN90-1.5, TN90-3.0, and TN90-Rubbing (a commercial twisted liquid-crystal cell, INSTEC, Boulder, CO, USA) with rubbed orientation films on both the top and bottom substrates and a cell gap of 3 μm. For a 90° twisted liquid-crystal cell, due to the limited anchoring energy on its top and bottom surfaces, the alignment direction of LC molecules at these surfaces will deviate from the grooves on the alignment layer by a certain angle Δ*φ*, as shown in Figure 5a. The actual twist angle of LC molecules *φ*_t_ = 90° − 2Δ*φ*. The closer *φ*_t_ is to 90°, the stronger the anchoring ability of the alignment layers. Using the twist balance method described in Ref. [39], we measured the actual twist angle ($\varphi_{t}$) and cell gap (*d*) of the samples, and the corresponding azimuthal anchoring energy ($W_{\varphi }$) could be calculated using the following Equation, and the results are given in Table 1:

(3) $$W_{\varphi }=\frac{2K_{22}\varphi_{t}}{dsin(2∆\varphi )}$$

The actual twist angle (88.9°) of the TN90-1.5 liquid-crystal cell is closest to 90°, demonstrating that the 1.5 μm periodic polymer grating has the strongest anchoring ability for LCs. Based on Equation (3), the calculated azimuthal anchoring energy is 1.82 × 10^−4^ J/m^2^, and it is larger than the commercial rubbed liquid-crystal cell (1.61 × 10^−4^ J/m^2^). Based on Equation (2), the theoretical anchoring energy of the alignment layer in the TN90-1.5 cell is calculated to be 0.61 × 10^−4^ J/m^2^. The experimental value is approximately three times as high as the theoretical value. This could be attributed to deeper grooves and slight periodic undulations on the top of the polymer, which may enhance the overall anchoring ability to liquid-crystal molecules. Additionally, the parabolic-like profile of polymer grating grooves deviates from the sinusoidal groove structure used in the Berreman model [28,33].

As shown in Figure 5b, compared with some commonly reported orientation techniques, such as rubbed orientation (~10^−4^ J/m^2^), photoalignment (~10^−4^ J/m^2^) [40], substrate patterning via optical interference (~1.43 × 10^−5^ J/m^2^) [41], and grating via DLW-TPP (~5×10^−6^ J/m^2^) [12], it is not difficult to find that the polymer grating structure prepared based on fractional Talbot lithography has the strongest anchoring performance. Furthermore, the resolution of molecular alignment is critical to realize the intricate LC alignment patterns. Nanoimprint lithography and DLW-TPP have the potential to align the liquid-crystal molecular with the high resolution beyond the micrometer scale [42,43], but they are expensive and inefficient for large area. In contrast, the resolution of photoalignment spans from micrometers to tens of millimeters [8], which is important to realize the intricate geometry patterns over a large area. The fractional Talbot lithography technique can not only maintain the advantage of large area alignment but also has a higher resolution than the photomask. That is to say, high-resolution periodic structures can be obtained with low-resolution photomasks at the fractional Talbot distance. This means minimum feature resolution below the micrometer level can also be obtained by using extreme ultraviolet light and submicron period photomasks [20,23], but the costs will also increase. In this work, we choose the micron-scale resolution, which greatly reduces costs comparing with the other alignment methods. It can be found that the lithography based on the fractional Talbot effect is more suitable for liquid-crystal orientation with the following advantages: (1) High-resolution polymer structures with micron scale can be obtained using low-resolution photomasks. This is important to realize the intricate geometry patterns over large areas with lower costs. Additionally, non-contact exposure makes it easier to obtain high-quality orientation structures, avoiding the introduction of impurity ions and facilitating mass production. (2) The anchoring energy strength is adjustable. The anchoring energy strength of the alignment layer in the TN90-3.0 cell is ~10^−5^ J/m^2^. For the TN90-1.5 cell, the enhanced anchoring energy (~1.82 × 10^−4^ J/m^2^) is consistent with the prediction of the Berreman theoretical model, where the anchoring energy increases with decreased period. The Berreman model provides a way to improve the anchoring energy of orientation layers by adjusting the slit width and the grating period of the photomask. (3) The LC alignment patterns are designable. Like other lithography techniques, the microstructures are decided by the photomask, and the designed photomask with a fractional Talbot effect can produce the corresponding LC alignment patterns.

### 3.2. Fractional Talbot Lithography for Large-Area Concentric Orientation of Liquid-Crystal Molecules

Special optical fields have attracted extensive attention from researchers due to their unique polarization and phase distributions. For instance, vortex beams, possessing an additional degree of freedom (orbital angular momentum), can be used in areas such as optical communications, quantum computing, quantum communications, and cryptography. Commonly, special optical fields can be generated using spiral phase plates, spatial light modulators, metasurfaces, and *q*-plates [44,45,46]. Due to the tunable properties of LCs, liquid-crystal *q*-plates hold significant promise for many applications. The typical LC *q*-plate consists of a nematic LC layer with a spatial in-plane director field distribution. The local spatial variation of the optical axis of the LC molecules is defined by:

(4) $$\alpha \left(\varphi \right)=q\varphi +\alpha_{0}$$

where the angle α describes the optical axis deviates from the *x*-axis, φ is the azimuthal angle, *q* is an integer standing for the defect strength of the *q*-plate, and $\alpha_{0}$ means the orientation of LC at the *x*-axis [13,47,48]. In this work, we have utilized this orientation technology to fabricate a liquid-crystal *q*-plate with *q* = 1 and $\alpha_{0}$ = π/2 to demonstrate its potential in designing non-uniform orientations of liquid-crystal molecules.

#### 3.2.1. Polarization Optical Microscopic Images of the *q*-Plate

**Figure 6:**
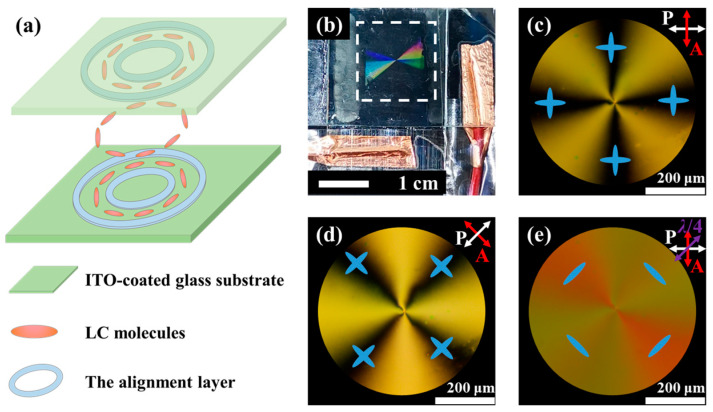
(**a**) Diagram of liquid-crystal cell and liquid-crystal molecule arrangement; (**b**) photo of the *q*-plate, with the white dashed box indicating the structural area; POM images under crossed polarizers with the polarization direction of (**c**) 0° and (**d**) 45°, respectively; (**e**) polarized light images after inserting a quarter-wave plate between the sample and the analyzer.

To assemble the *q*-plate (*q* = 1 and $\alpha_{0}$ = π/2) using alignment layers with a good anchoring ability, a 2D concentric ring polymer structure was designed, where the height and period of the concentric rings were kept consistent with the previous 1D grating structure. After the exposure process, the 2D concentric ring polymer film was fabricated on an ITO-coated glass substrate. A liquid-crystal cell was assembled using two substrates with a 2D concentric ring polymer film according to the configuration shown in Figure 6a, and then it was filled with LCs to form a *q*-plate. The cell gap was 10 μm. Figure 6b shows a photo of the *q*-plate, which exhibits dazzling colors under sunlight, indicating its well-defined periodic structure. By using a cross-polarized optical microscope, a perfect Maltese cross-extinction phenomenon was observed, as shown in Figure 6c,d. As the crossed polarizers were rotated simultaneously, the extinction position of the liquid-crystal rotated accordingly. It is not difficult to discover the reason for this phenomenon: in the extinction region, the optical axis of the liquid-crystal molecules is perpendicular or parallel to the polarization direction of the incident linearly polarized light (as shown in Figure 6c,d, with the blue ellipses indicating the possible directions of LC molecules), which does not alter the polarization state of the incident light. Therefore, these regions appear dark. To determine the actual orientation of the liquid-crystal molecules, a quarter-wave plate (QWP, 532 nm) was inserted between the sample and the analyzer. Different interference color changes were observed in the four bright regions compared with Figure 6c, where LCs with directors parallel to the slow axis of the QWP became red in the second and fourth quadrants, and LCs with directors perpendicular to the slow axis of the QWP became greenish in the first and third quadrants [13,49]. This means that the liquid-crystal molecules orient azimuthally, as shown by the blue ellipses in Figure 6e. This azimuthal orientation pattern confirms that we have successfully fabricated a liquid-crystal *q*-plate with *q* = 1 and $\alpha_{0}$ = π/2.

#### 3.2.2. Electrical Tunability of Liquid-Crystal *q*-Plate

**Figure 7:**
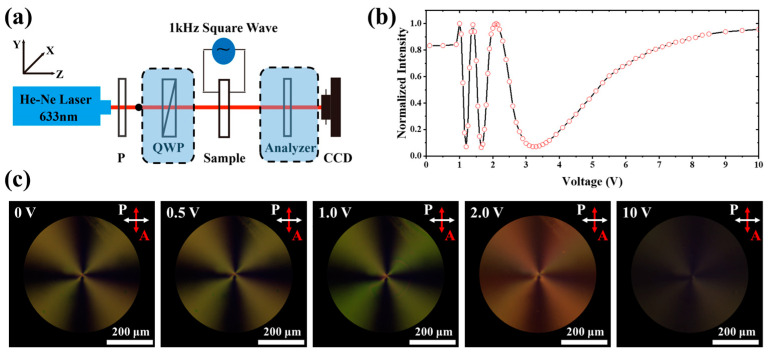
(**a**) The setup for generating and analyzing special optical fields; the components within the dashed boxes can be changed to realize different functions; (**b**) normalized transmitted light intensity through a *q*-plate measured at different voltages; (**c**) POM images under different voltages: 0 V, 0.5 V, 1.0 V, 2.0 V, and 10 V.

The experimental setup in Figure 7a was used to study the electrical tuning capability and light field modulation ability of the prepared *q*-plate. Linearly polarized light at 633 nm was converted into left-handed circularly polarized light after passing through a quarter-wave plate. When the circularly polarized light was incident on the *q*-plate, the transmitted light field can be expressed using a Jones matrix as follows [47]:

(5) $$E_{out}=\cos\left(\frac{\delta }{2}\right)\times \frac{1}{\sqrt{2}}\left(\begin{array}{c} 1\\ -i \end{array}\right)+i\sin\left(\frac{\delta }{2}\right)\times e^{-i2\alpha }\times \frac{1}{\sqrt{2}}\left(\begin{array}{c} 1\\ i \end{array}\right)$$

where *δ* represents the phase retardation generated by the *q*-plate, and for our *q*-plate, α=φ+π/2. When a left-handed circular analyzer was put behind the sample to select the left-handed component, the emergent light intensity was proportional to cos^2^(*δ*/2). Therefore, by applying a square wave with a repeated frequency of 1 kHz to the liquid-crystal cell and monitoring the change in light intensity with a power meter, we obtained the relation between the light intensity and voltage, as shown in Figure 7b. When the voltage increases, the intensity experiences an oscillation, and the maximum values correspond to even multiples of the phase retardation of a half wavelength, while the minimum values correspond to odd multiples. This curve indicates that the assembled liquid-crystal cell has a very low driving voltage (~1 V) and flexible electrical tuning capability, achieving a 6π phase retardation adjustment within a 5 V range, suggesting its potential for wide phase tuning. Figure 7c shows the POM images under different voltages; when the voltage increases from 0 V to 10 V gradually, the color of the pattern begins to change at 1 V; this low voltage is very usual for commercial LC mixtures. The different colors indicate the retardation change caused by the reorientation of LC molecules under the different applied voltages. When the voltage increases to 10 V, the cross-pattern almost disappears, indicating a homeotropic orientation of LC molecules.

#### 3.2.3. Generation of Special Optical Fields

**Figure 8:**
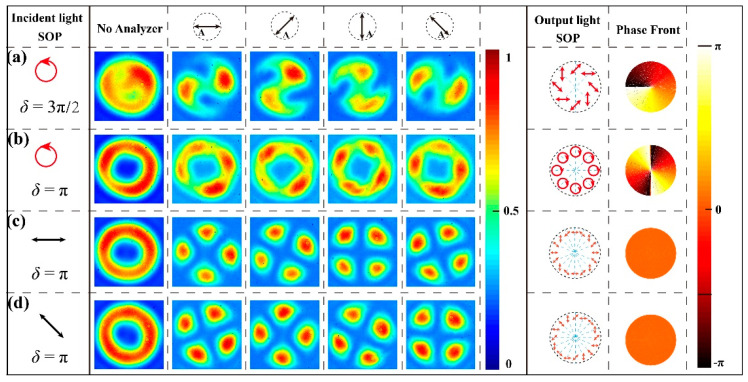
Generated beam profiles detected with/without analyzer for different optical retardations and incident light: (**a**) *δ* = 3π/2, LCP; (**b**) *δ* = π, LCP; (**c**) *δ* = π, LP along *x*-axis; and (**d**) *δ* = π, LP with −45°.

From an application perspective, there are two particularly interesting cases for liquid-crystal *q*-plates, namely when the phase retardations are 3π/2 (or π/2) and π, respectively. When the incident light is left-handed circularly polarized (LCP) and the phase retardations of the *q*-plate are set to 3π/2 and π, respectively, the Jones matrices for the emergent light fields can be calculated according to Equation (5). The state of polarizations (SOP) and phase fronts are shown in Figure 8 (Columns 7 and 8). As shown in Figure 8a (*δ* = 3π/2), the transmitted light intensity distribution exhibits a unique pattern with a weak center and strong edges. When adding an analyzer between the sample and CCD, an S-shaped pattern emerges, and the extinction positions are rotated with the analyzer. This phenomenon indicates that the special optical field has an axially symmetric spatial polarization distribution. As illustrated in Figure 8b (*δ* = π), the emergent light field is an optical vortex with a helical phase front, and the phase singularity at the center results in a hollow ring-shaped distribution, with the central intensity being zero. When rotating the analyzer, the observed spot remains a bright ring, which indicates that the light beam is circularly polarized.

As shown in Figure 8c,d, the phase retardation is fixed to π, linearly polarized (LP) incident light will be converted to a hollow spot due to the presence of a polarization singularity, and the shape of the emergent spot remains unchanged when rotating the polarization direction of the incident light. After adding an analyzer between the sample and CCD, there are four extinction positions, where the directions of the polarization are perpendicular to the transmission axis of the analyzer. As the analyzer rotates, the extinction positions rotate in the same direction but with a slow speed, which proves that any position on the cross-section of the light beam is linearly polarized, and the beam possesses rotational symmetry. By adjusting the applied voltage and changing the polarization state of the incident light, it is possible to simultaneously modulate both the polarization state and the phase of the incident light. Thus, we can generate vortex beams and axially symmetric vector beams. This method fully demonstrates that the lithography technology based on the fractional Talbot effect can not only achieve uniform large-area processing but can also meet the demand for patterned orientation.

## 4. Conclusions

In this paper, we introduce a cost-effective liquid-crystal alignment method by utilizing fractional Talbot lithography. Based on the Talbot effect, 1D grating and concentric ring polymer films with a period of 1.5 μm are obtained at the half Talbot distance. The 1D polymer grating alignment layers can uniformly align liquid-crystal molecules over large areas and achieve anchoring energy as strong as 1.82 × 10^−4^ J/m^2^. For the 2D concentric ring alignment layers, a non-uniform alignment of LCs is achieved, and a Maltese cross-pattern can be observed using a polarized optical microscope under a crossed configuration. Meanwhile, by adjusting the polarization state and phase retardation of the incident light, special optical fields such as axially symmetric vector beams and vortex beams can be generated. This technique ensures uniform alignment across extensive areas and offers precise control over non-uniform patterned liquid-crystal alignment by customizing photomasks, which may have promising applications in other types of LCs or alignment-sensitive materials.

## Figures and Tables

**Table 1:** The anchoring energy of three different liquid-crystal cells.

Cell	Cell Gap *d* (μm)	Actual Twist Angle *φ*_t_ (deg)	Anchoring Energy *W_φ_* (J/m^2^)
TN90-Rubbing	3.00	88.0	1.61 × 10^−4^
TN90-1.5	4.88	88.9	1.82 × 10^−4^
TN90-3.0	5.06	71.9	0.88 × 10^−5^

## Data Availability

The original contributions presented in the study are included in the article; further inquiries can be directed to the corresponding author.
